# Supplementary material for: Solid solution for catalytic ammonia synthesis from nitrogen and hydrogen gases at 50 °C
Source: Nat Commun. 2020 Apr 24;11:2001. doi: 10.1038/s41467-020-15868-8 (PMC7181780; doi:10.1038/s41467-020-15868-8)
Supplement: Supplementary file 1 — Supplementary Information [file 41467_2020_15868_MOESM1_ESM.pdf]

Supplementary Information

Solid solution for catalytic ammonia synthesis from nitrogen and hydrogen gases at 50 °C

Hattori et al.

Supplementary Discussion

Electron donation from transition metals to adsorbed molecules

The electron-donating capability of TM surfaces can be estimated by measuring the wavelength of CO adsorbed on TM surfaces. The wavelength of CO stretching is generally red-shifted, and the intensity of the CO stretching band is decreased with an increase in the temperature[1,2]. This cannot be attributed to an increase in back-donation from the TM to adsorbed CO but to a decrease in adsorbed CO due to CO desorption with an increase in the temperature.

N species formed on catalysts by activation at 340 °C

In this study, low temperature ammonia synthesis was carried out through ammonia synthesis in a flow of N_2−H_2 at 340 °C, followed by cooling down below 20 °C in a flow of N_2. It was confirmed by XPS measurements that the prepared Ru/CaFH samples had no surface N species. Moreover, when the tested catalysts, including Ru/CaFH, cooled down below 20 °C were heated in a flow of H_2 or He at 1 °C min^{-1}, the desorption of ammonia and N_2 was not detected at all even by a mass spectrometer.

CWEY at each pressure

It is desirable that all chemicals be produced in yields close to the equilibrium yields. The energy consumption of a reactor filled up with catalyst becomes large in proportion to the amount of catalyst necessary for an equilibrium product yield. As a result, CWEY can be an index for ammonia production efficiency from the viewpoints of kinetics, thermodynamics and practice. Furthermore, the catalytic activities of different catalysts under different pressures at the same temperature can be roughly compared with each other according to the CWEY. For ammonia synthesis over the commercial Fe catalyst at 400 °C, the CWEY under 0.9 MPa is only 1.3 times larger than that at 0.1 MPa, although the former reaction pressure is 9 times higher than the latter[3]. Thus, the CWEY at higher pressure is not significantly difference from that at lower pressure, although the CWEY tends to increase with the total pressure; the catalytic activities of different catalysts under different pressures can be roughly compared with each other according to the CWEY. CWEYs were estimated from the rates of ammonia formation at each reaction temperature. It was confirmed that the rates of ammonia formation (μmol h^{-1}) for Ru/CaFH, Ru/BaO-BaH_2 and Ru/CaH_2 increased in direct proportion to the catalyst weight (0.05-5.00 g).

Supplementary Figures

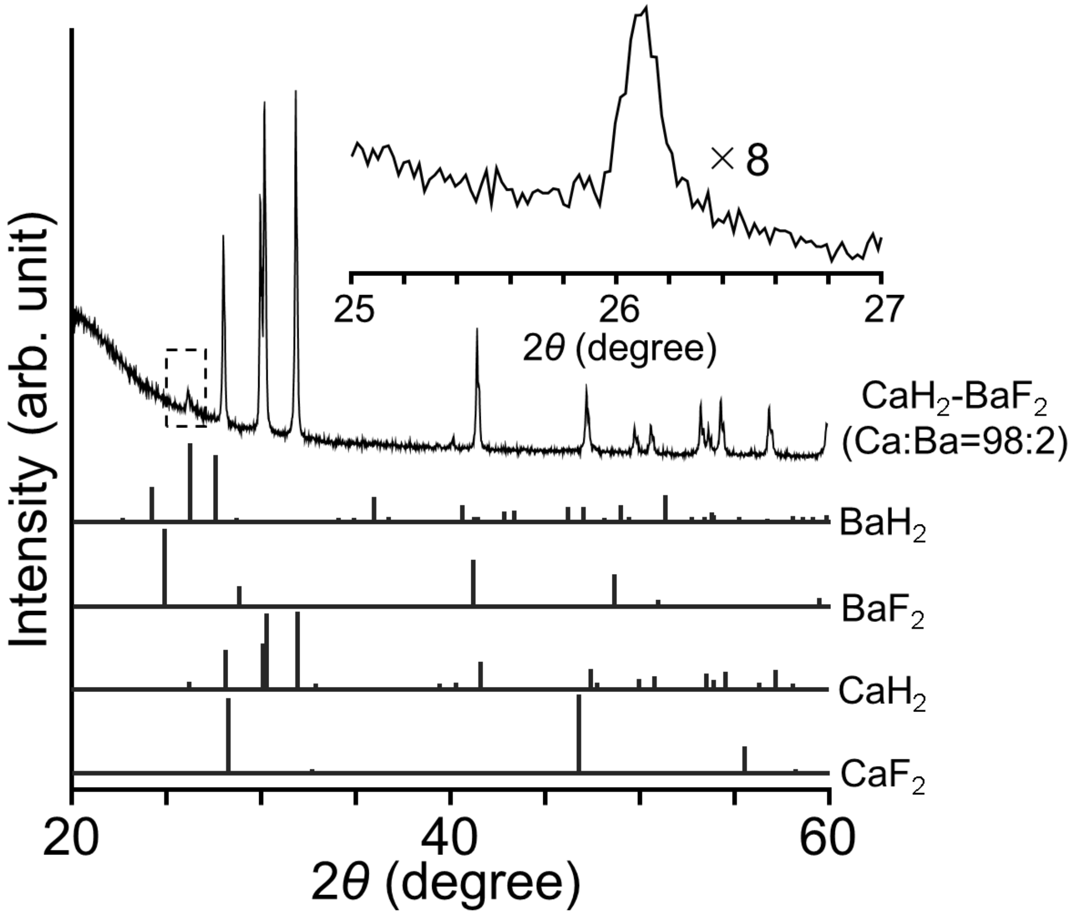

Supplementary Fig. 1 | XRD pattern for CaH_2-BaF_2 mixture (Ca:Ba=98:2). For comparison, the XRD patterns for BaH_2, BaF_2, CaH_2 and CaF_2 are also shown in this figure.

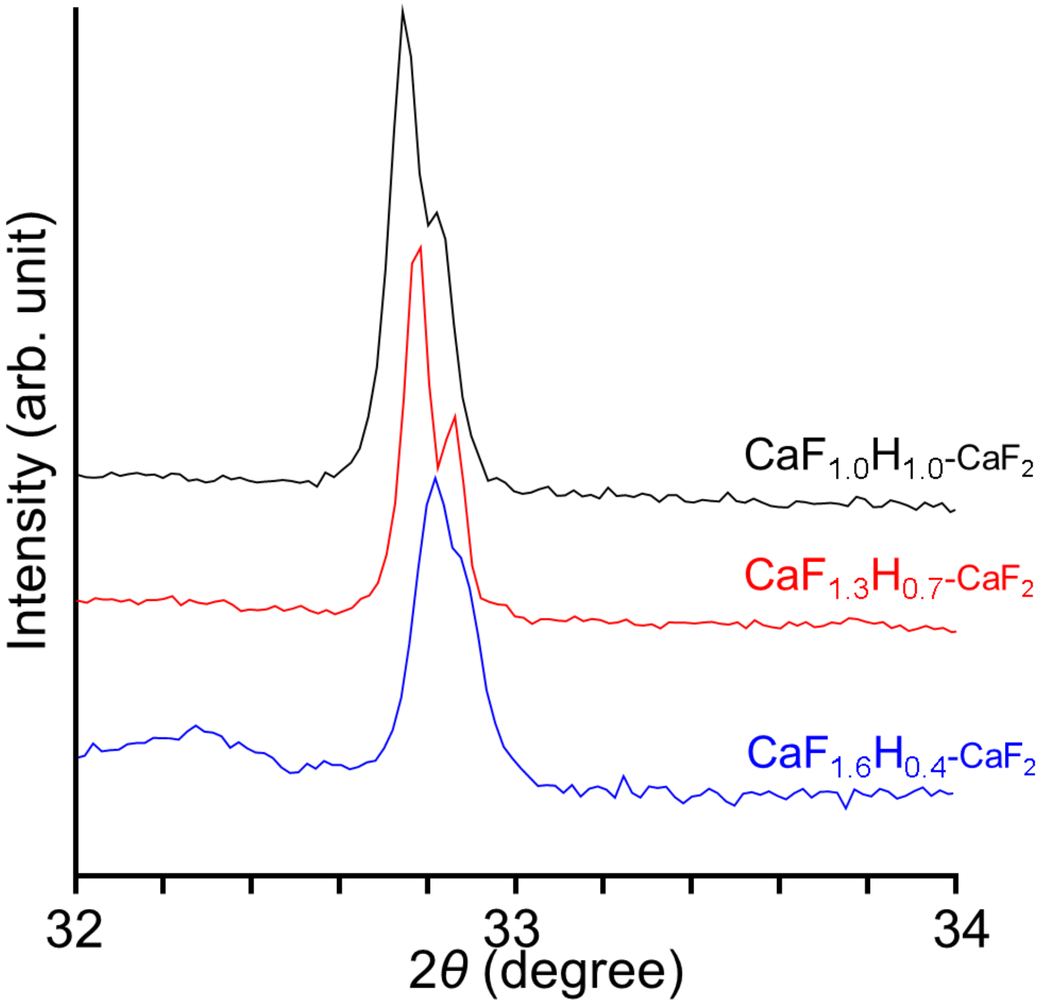

Supplementary Fig. 2 | XRD patterns for CaF_xH_{2-x}-CaF_2 ($1.0 \leq x \leq 1.6$).

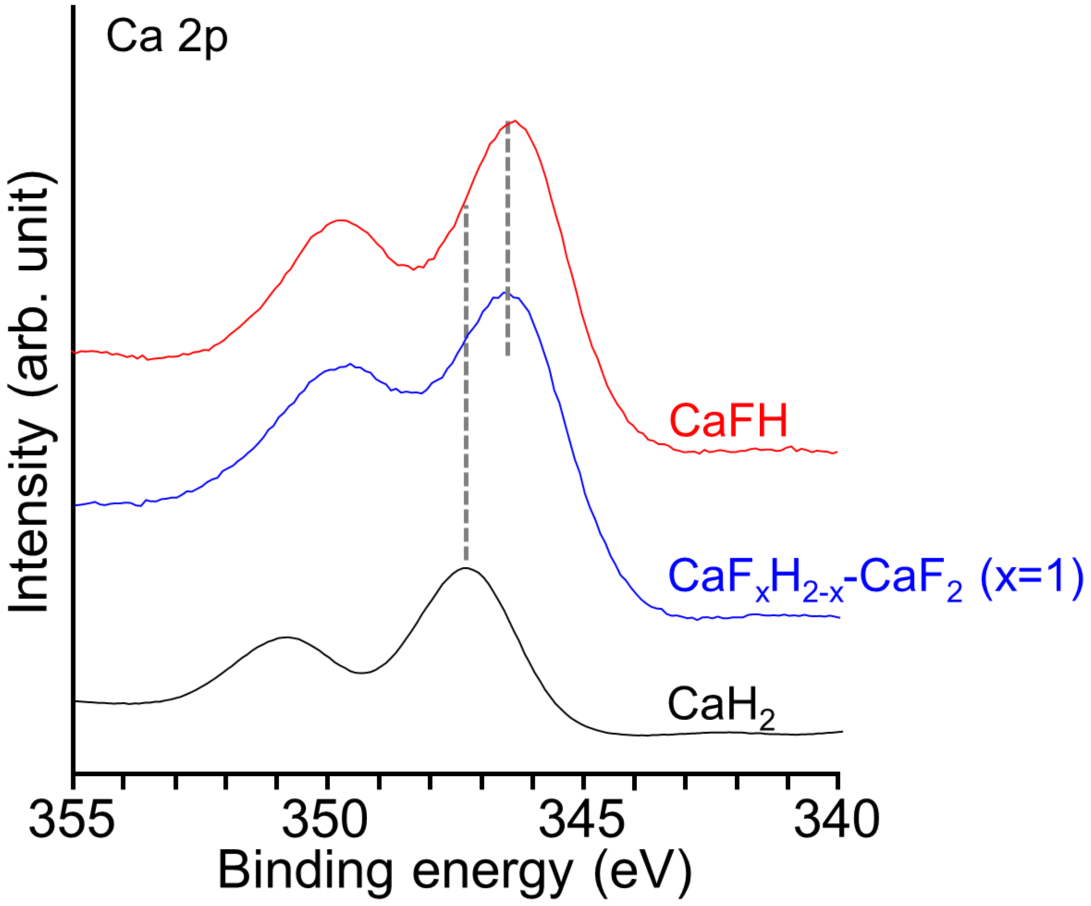

Supplementary Fig. 3 | Ca 2p XPS for CaH_2-BaF_2, CaF_xH_{2-x}-CaF_2 and CaH_2.

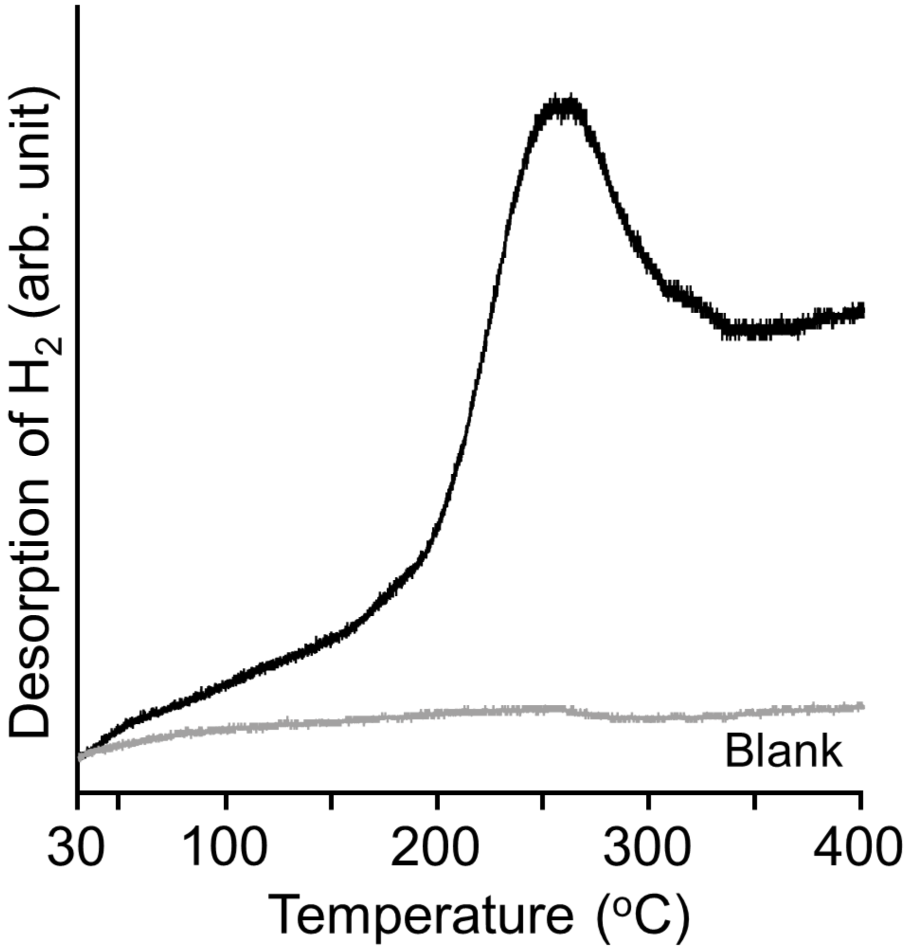

Supplementary Fig. 4 | H_2-TPD profile for Ru/CaF_xH_{2-x}-CaF_2 (x=1). The H_2-TPD profile for Ru (12 wt%)/CaF_xH_{2-x}-CaF_2 (x=1) after ammonia synthesis reaction at 340 °C, followed by cooling down below 20 °C. TPD measurements were performed under Ar flow (1 °C min^{-1}). No desorption of molecular species giving the *m*/*z*=2 signal, such as H_2O, was observed.

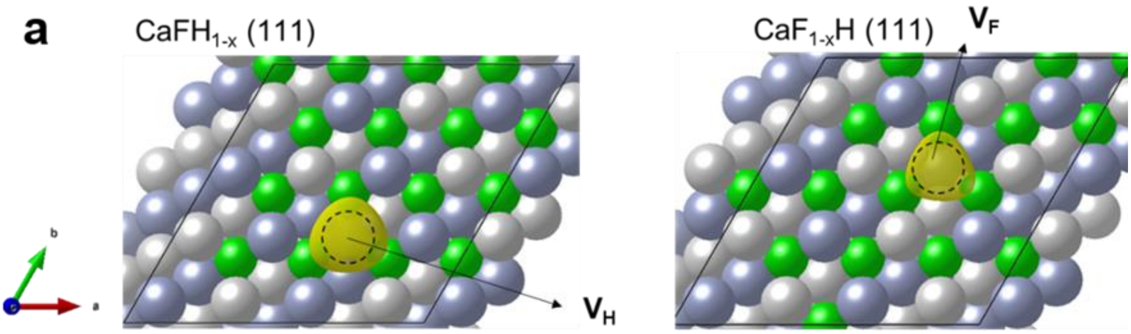

b

Miller index	Surface energy (J m^{-2})
111	0.34
101	0.55

c

Miller index	Defect	Work function (eV)
111	H	2.2
111	F	2.2

Supplementary Fig. 5 | DFT results for CaFH with F^- or H^- defects. a, The surface model of CaFH solutions with H^- and F^- defects. **b,** DFT calculations revealed that the (111) surface is the most stable in the cubic CaFH solid solution, which is consistent with the (111) surface also being the cleavage plane of cubic CaF_2.**c,** The work function of CaFH solutions with H^- and F^- defects (-1.0 < EF < 0.0, isosurface = 0.002) that trap electrons was estimated to be 2.2 eV, and there was no difference in the work functions between both cases with either defects.

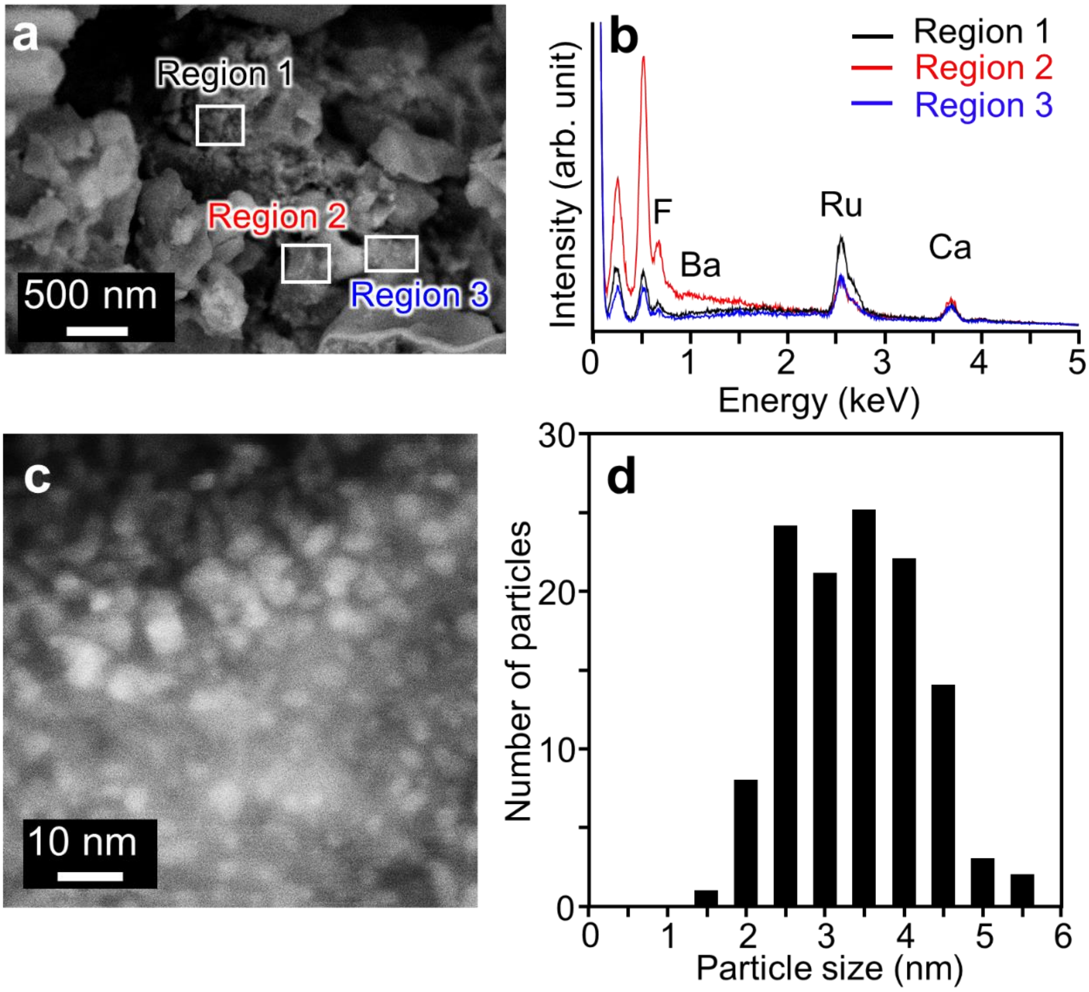

Supplementary Fig. 6 | Morphological information for Ru/CaFH. a, Backscattering electron (BSE) image of Ru/CaFH after ammonia synthesis for 50 h. **b,** EDX spectra for each region in a. **c,** STEM image of Ru/CaFH after ammonia synthesis for 50 h. **d,** Particle size distributions of Ru/CaFH. The number of particles measured from STEM images was more than 100.

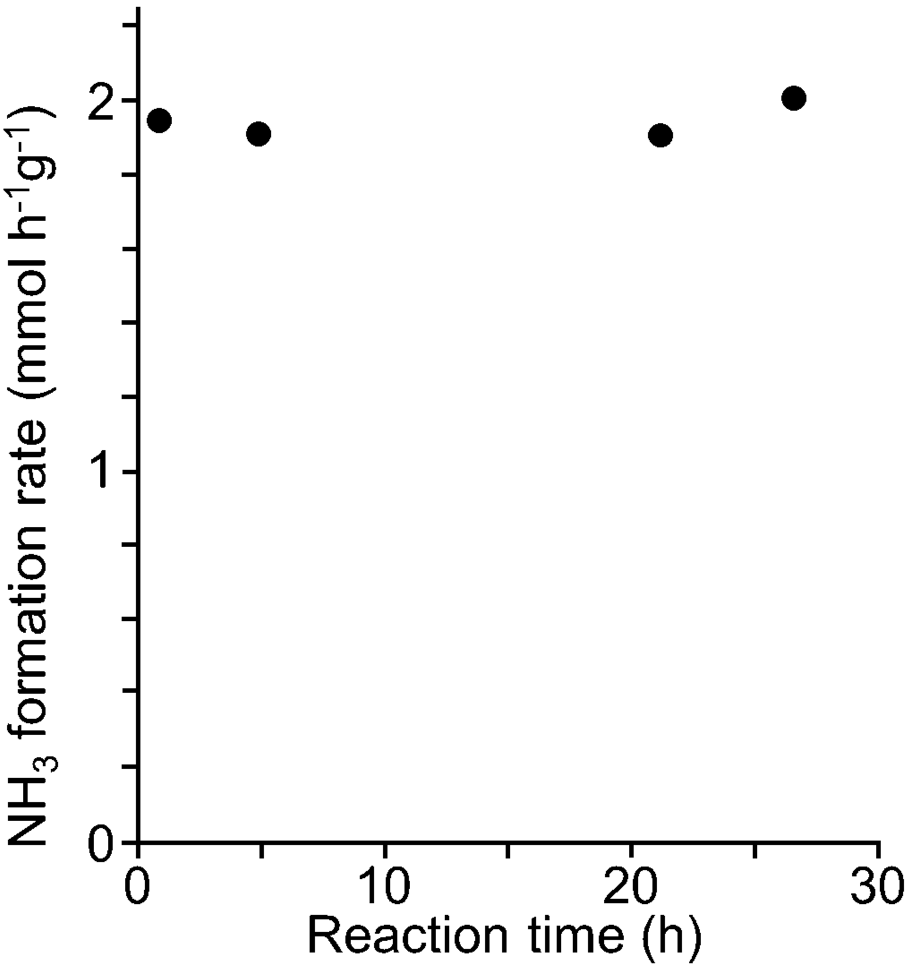

Supplementary Fig. 7 | Ammonia synthesis over Ru/CaFH at 200 °C. Time courses for ammonia synthesis over Ru/CaFH catalyst (reaction conditions: catalyst (0.05 g), synthesis gas (N_2/ H_2 = 1:5, 60 mL min^{-1}), temperature (200 °C), pressure (0.1 MPa)).

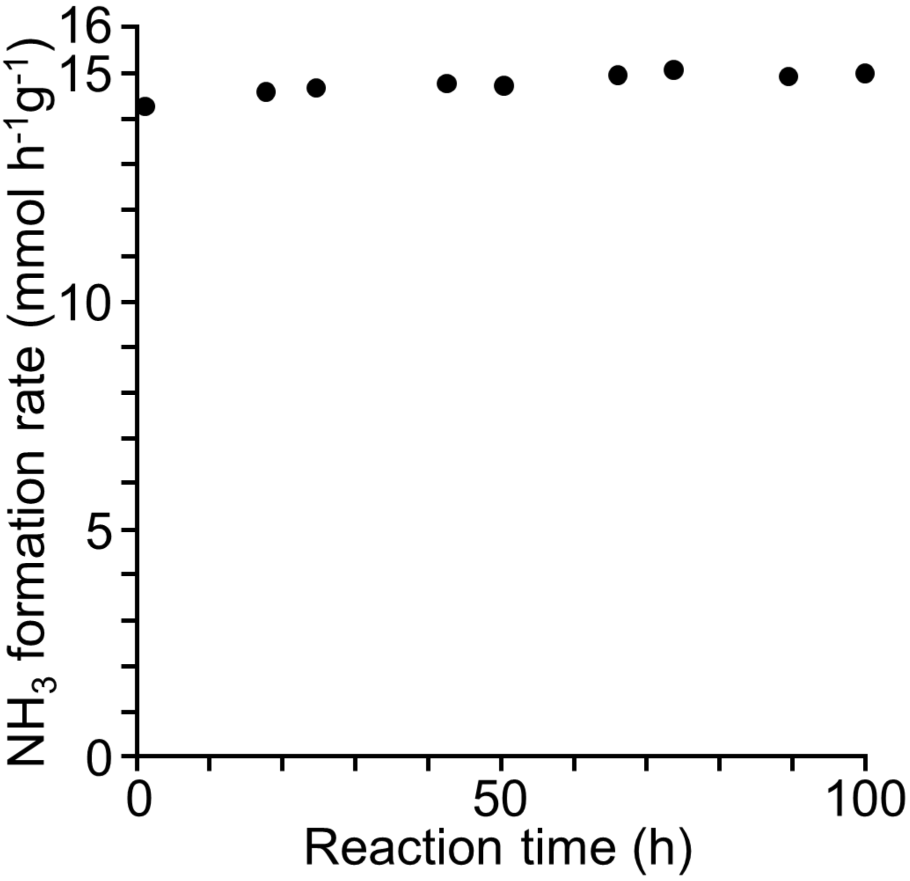

Supplementary Fig. 8 | Ammonia synthesis over Ru/CaFH at 340 °C. Time courses for ammonia synthesis over Ru/CaFH catalyst (reaction conditions: catalyst (0.10 g), synthesis gas (N_2/ H_2 = 1:3, 60 mL min^{-1}), temperature (340 °C), pressure (0.1 MPa)). The amount of ammonia produced by Ru/CaFH at 340 °C exceeded the amount of the used catalyst (ca. 24 mmol) within 100 min.

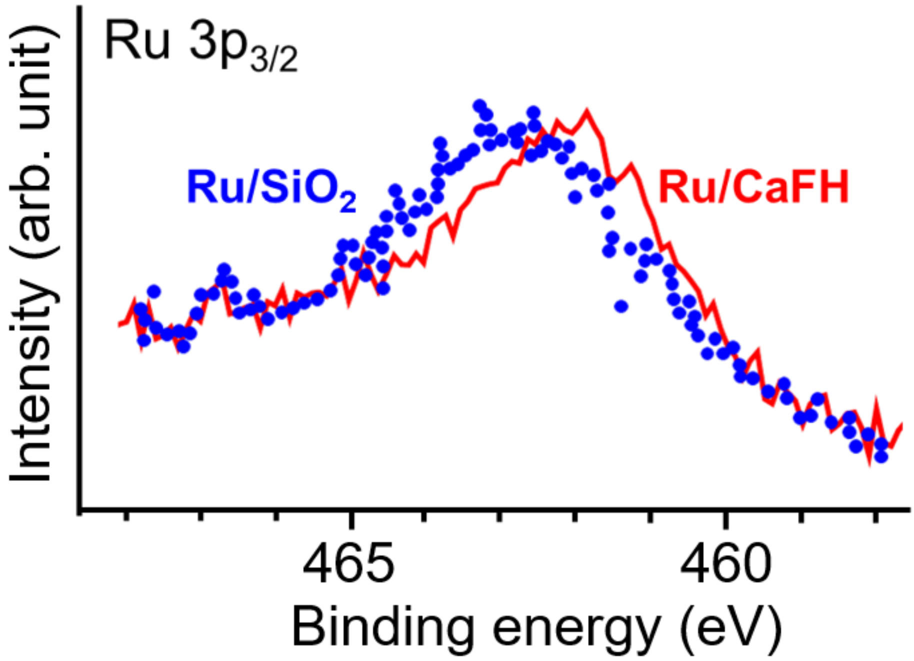

Supplementary Fig. 9 | XPS Ru 3p3/2 spectra of Ru/CaFH and Ru/SiO_2. 10 wt% Ru/SiO_2 was prepared by an impregnation method. 2 g of SiO_2 (surface area: 30 m^2 g^{-1}) was initially dispersed into an aqueous solution (50 mL) containing Ru(NO)$(NO_3)_3$, which corresponds to 10 wt% Ru, with stirring and kept overnight. The mixed solution was slowly evaporated at 333 K under 0.01 MPa and then dried at 373 K for 1 h under vacuum conditions. The recovered solid powder was pretreated under 5%H_2/Ar flow (50 mL min^{-1}) at 673 K for 2 h. The average Ru particle size of 10 wt% Ru/SiO_2 was 3.2 nm. This was close to that of Ru/CaFH (3.4 nm).

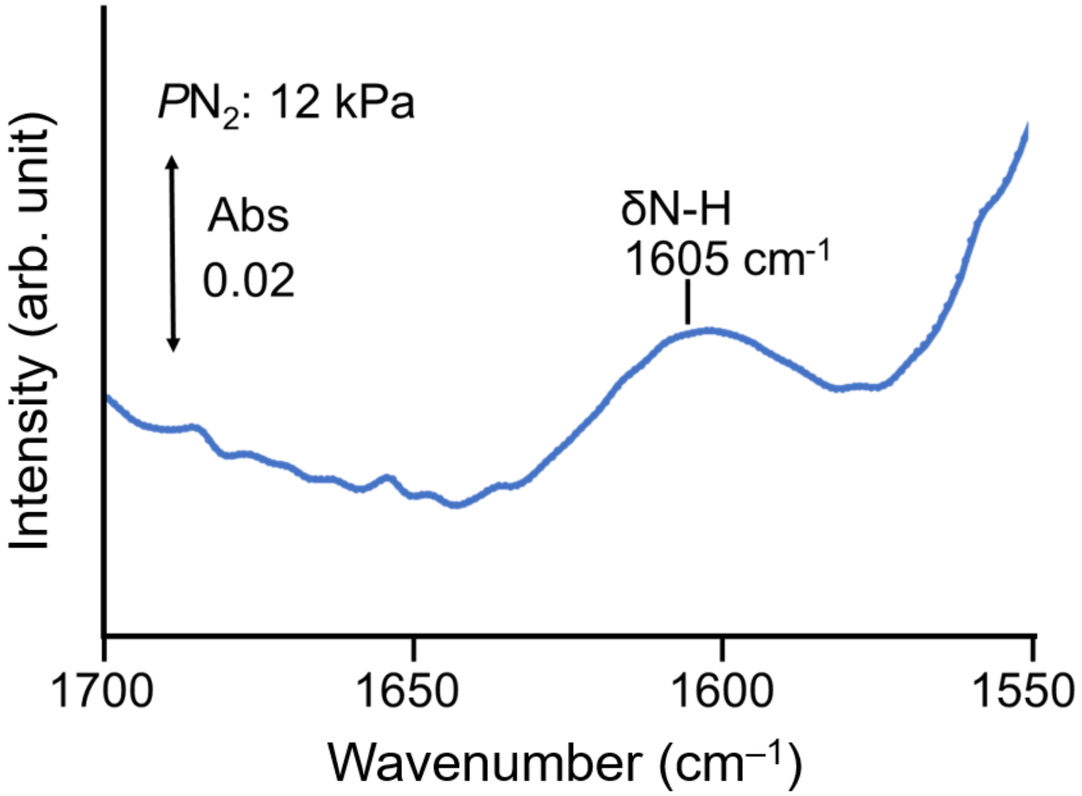

Supplementary Fig. 10 | FT-IR spectra for N_2-adsorbed Ru/CaFH. FT-IR spectra for N_2 adsorption on Ru/CaFH after ammonia synthesis reaction at 340 °C, followed by cooling down below 20 °C. N_2 was adsorbed on the catalysts at 25 °C in the presence of N_2 (12 kPa). The δNH bending band was not observed under vacuum. N_2 adsorption on Ru/CaFH prepared in a flow of pure H_2 gas alone (see discussions on above section) was also examined by FT-IR. There was no difference in FT-IR spectrum between Ru/CaFH catalysts prepared with N_2−H_2 and those with H_2 alone.

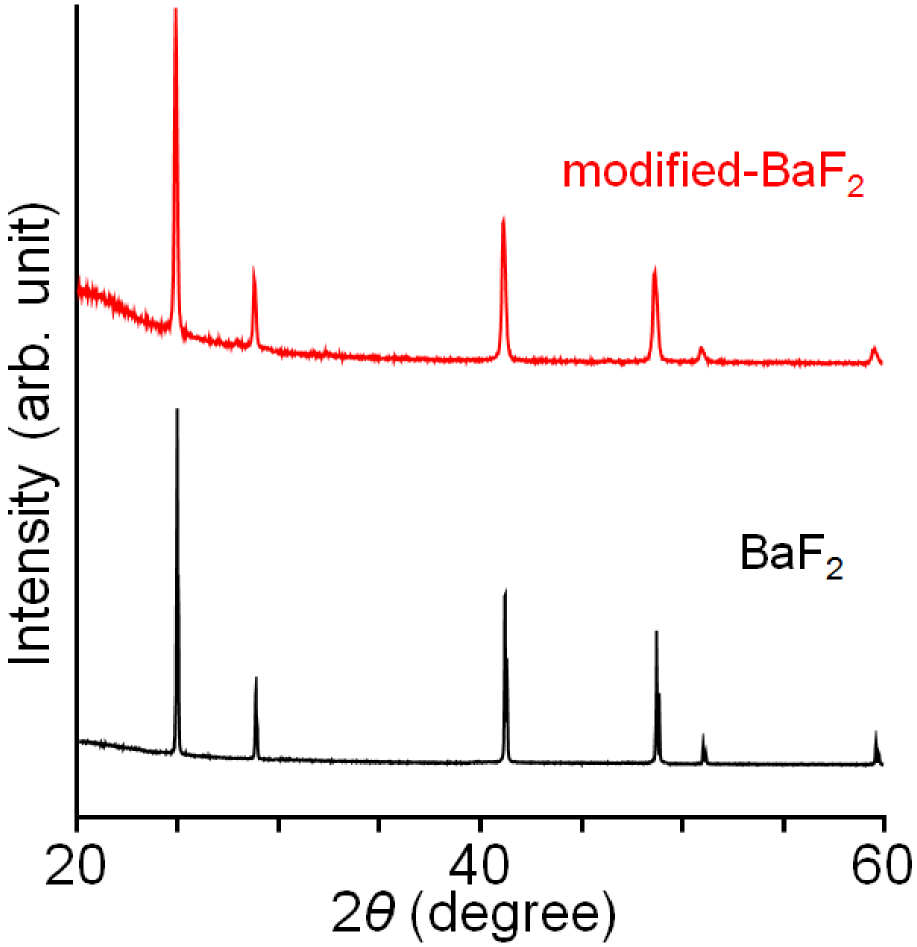

Supplementary Fig. 11 | XRD patterns. XRD patterns for BaF_2 and modified-BaF_2.

Supplementary Tables

Supplementary Table 1 | Ammonia synthesis over various Ru catalysts. Difference between the measured and estimated rates of ammonia formation over various Ru-deposited catalysts.

Catalysts	Temperature (°C)	$r^{E}_{NH_3}$ (μmol g^{-1} h^{-1})	$r^{M}_{NH_3}$ (μmol g^{-1} h^{-1})
Ru (10%)/CaH_2	300	3200	3200
	200	150	80
	150	20	—
Ru (10%)/BaO-BaH_2	300	6800	6800
	200	640	420
	150	130	40
	100	20	—
Ru (10%)-Cs/MgO	300	2340	2340
	200	170	90
	150	—	—
Ru (2%)/C12A7	300	1153	1150
	200	160	—

Reaction conditions: catalyst (0.1 g), synthesis gas (H_2/N_2 = 3/1, 60 mL min^{-1}), pressure (0.1 MPa).

Supplementary Table 2 | Ammonia synthesis over various catalysts.

Catalysts	S_{BET} (m^2 g^{-1})	Pore size (nm)	Ru particle size (nm)	Temperature (°C)	rNH_3 (μmol g^{-1} h^{-1})
Ru (12%)/CaFH	30	-	3.4 [a]	340	15000
				300	12900
				200	1250
				100	120
Ru (10%)/CaH_2	13	-	2.7 [a]	340	7400
				300	3200
				200	80
				100	-
Ru (10%)/Ba-$Ca(NH_2)_2$	101	3-30	2.7 [a]	340	12400
				300	9100
				200	630
				100	-
Ru (10%)/BaO-BaH_2	20	-	4.0 [a]	340	10500
				300	6800
				200	420
				100	-
Ru (2%)/C12A7	1	-	28.7 [b]	340	2000
				300	1150
				200	-
Cs-Ru (10%)/MgO	12	-	5.2 [a]	340	8320
				300	2340
				200	90
				100	-
commercial Fe catalst	-	-	-	340	1740
				300	1220
				200	-

Ru particle size was estimated (a) by averaging the particle size distribution measured using STEM and (b) from active site numbers determined by the CO pulse chemisorption method (Ru/CO = 1). NH_3 synthesis rate (r_{NH_3}); reaction conditions: catalyst (0.1 g), synthesis gas (H_2/N_2 = 3, 60 mL min^{-1}), pressure (0.1 MPa).

Supplementary Table 3 | CWEYs for various catalysts.

Catalysts	Temperature (°C)	Pressure (MPa)	WHSV (mL g^{-1} h^{-1})	CWEY (g)	r_{NH_3} (μmol g^{-1} h^{-1})	Ref.
Ru (12%)/CaFH	340	0.1	36000	1.0	15000	This work
	300	0.1	36000	2.4	12900	
	200	0.1	36000	150	1250	
Ru (10%)/CaH_2	340	0.1	36000	2.1	7400	This work
	300	0.1	36000	10	3200	
	200	0.1	36000	1370	80	
Ru (10%)/BaO-BaH_2	340	0.1	36000	1.5	10500	This work
	300	0.1	36000	4.6	6800	
	200	0.1	36000	440	420	
Ru (10%)/Ba-$Ca(NH_2)_2$	340	0.1	36000	1.2	12400	This work
	300	0.1	36000	3.4	9100	
	200	0.1	36000	300	630	
Cs-Ru (10%)/MgO	340	0.1	36000	1.8	8320	This work
	300	0.1	36000	13	2300	
	200	0.1	36000	1920	100	
commercial Fe catalyst	340	0.1	36000	8.9	1740	This work
	300	0.1	36000	25	1220	
	200	0.1	36000	-	-	
Fe-LiH	350	1.0	60000	17	10500	4
	300	1.0	60000	77	4300	
	200	1.0	60000	1820	450	
Co-LiH	350	1.0	60000	16	11200	4
	300	1.0	60000	71	4700	
	200	1.0	60000	2050	400	
BaH_2-Co/CHTs	350	1.0	60000	15	11600	5
	300	1.0	60000	64	5200	
	200	1.0	60000	1780	460	
Ru/$La_{0.5}Ce_{0.5}O_{1.75}$	300	1.0	72000	40	10700	6
Ru/$La_{0.5}Pr_{0.5}O_{1.75}$	300	1.0	72000	49	8900	7

Supplementary Table 4 | Activities over Ru/CaF_xH_{2-x}-CaF_2 and Ru/BaH_2.

Catalysts	Surface area of support (m^2 g^{-1})	r_{NH_3} (µmol g^{-1} h^{-1})
Ru (12%)/CaFH	10	15000
Ru (12%)/CaF_xH_{2-x}-CaF_2 (x=1)	1	7000
Ru (10%)/BaH_2	1	200

Reaction conditions: catalyst (0.1 g), synthesis gas (H_2/N_2 = 3, 60 mL min^{-1}), temperature (340 °C), pressure (0.1 MPa).

Supplementary References

1. Harle, H., Mendel, K., Metka, U., Volpp, H. R., Willms, L. & Wolfrum, J. Temperature dependence (90-440 K) of the vibrational spectra of CO adsorbed on platinum (111) studied by sum-frequency generation. *Chem. Phys. Lett.* **279**, 275–281 (1997).
2. Carlsson, A. F., Baumer, M., Risse, T. & Freund, H. J. Surface structure of Co–Pd bimetallic particles supported on thin films studied using infrared reflection absorption spectroscopy of CO. *J. Chem. Phys.* **119**, 10885–10894 (2003).
3. Kitano, M. et al. Self-organized ruthenium-barium core-shell nanoparticles on a mesoporous calcium amide matrix for efficient low-temperature ammonia synthesis. *Angew. Chem.* **130**, 2678–2682 (2018).
4. Wang, P. K. et al. Breaking scaling relations to achieve low-temperature ammonia synthesis through LiH-mediated nitrogen transfer and hydrogenation. *Nat. Chem.* **9**, 64–70 (2017).
5. Gao, W. B. et al. Barium hydride-mediated nitrogen transfer and hydrogenation for ammonia synthesis: a case study of cobalt. *ACS Catal.* **7**, 3654–3661 (2017).
6. Ogura, Y. et al. Efficient ammonia synthesis over a Ru/La0.5Ce0.5O1.75 catalyst pre-reduced at high temperature. *Chem. Sci.* **9**, 2230–2237 (2018).
7. Ogura, Y. et al. Ru/La0.5Pr0.5O1.75 catalyst for low-temperature ammonia synthesis. *ACS Sustainable Chem. Eng.* **6**, 17258–17266 (2018).
